# Immunometabolic adaptation and immune plasticity in pregnancy and the bi-directional effects of obesity

**DOI:** 10.1093/cei/uxac003

**Published:** 2022-02-24

**Authors:** April Rees, Oliver Richards, Megan Chambers, Benjamin J Jenkins, James G Cronin, Catherine A Thornton

**Affiliations:** Institute of Life Science, Swansea University Medical School, Swansea, Wales SA2 8PP, UK; Institute of Life Science, Swansea University Medical School, Swansea, Wales SA2 8PP, UK; Institute of Life Science, Swansea University Medical School, Swansea, Wales SA2 8PP, UK; Institute of Life Science, Swansea University Medical School, Swansea, Wales SA2 8PP, UK; Institute of Life Science, Swansea University Medical School, Swansea, Wales SA2 8PP, UK; Institute of Life Science, Swansea University Medical School, Swansea, Wales SA2 8PP, UK

**Keywords:** obesity, pregnancy, immunometabolism, plasticity

## Abstract

Mandatory maternal metabolic and immunological changes are essential to pregnancy success. Parallel changes in metabolism and immune function make immunometabolism an attractive mechanism to enable dynamic immune adaptation during pregnancy. Immunometabolism is a burgeoning field with the underlying principle being that cellular metabolism underpins immune cell function. With whole body changes to the metabolism of carbohydrates, protein and lipids well recognised to occur in pregnancy and our growing understanding of immunometabolism as a determinant of immunoinflammatory effector responses, it would seem reasonable to expect immune plasticity during pregnancy to be linked to changes in the availability and handling of multiple nutrient energy sources by immune cells. While studies of immunometabolism in pregnancy are only just beginning, the recognised bi-directional interaction between metabolism and immune function in the metabolic disorder obesity might provide some of the earliest insights into the role of immunometabolism in immune plasticity in pregnancy. Characterised by chronic low-grade inflammation including in pregnant women, obesity is associated with numerous adverse outcomes during pregnancy and beyond for both mother and child. Concurrent changes in metabolism and immunoinflammation are consistently described but any causative link is not well established. Here we provide an overview of the metabolic and immunological changes that occur in pregnancy and how these might contribute to healthy versus adverse pregnancy outcomes with special consideration of possible interactions with obesity.

## Teaser

Metabolic and immune adaptation are both features of pregnancy. The underlying principle of immunometabolism is that cellular metabolism determines fate and function of immune cells. Immunometabolism might be a key determinant of immune plasticity in pregnancy and modified by obesity for adverse pregnancy outcome.

## Introduction

**Figure 1:**
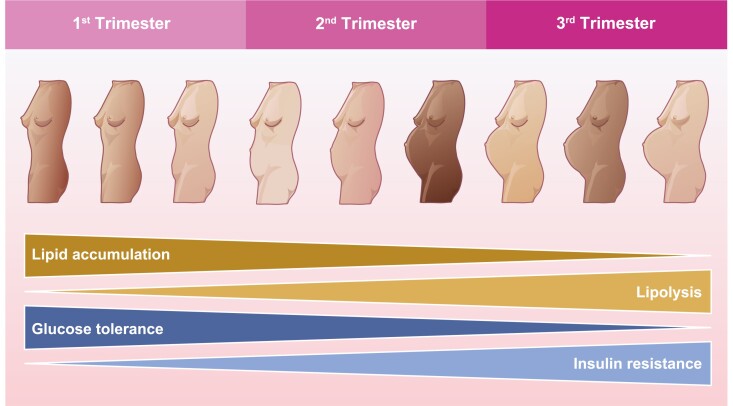
The key whole body metabolic changes that occur as pregnancy progresses. The accumulation of lipids decreases with pregnancy while lipolysis increases. The mother becomes less tolerant to glucose and insulin resistance is induced.

To meet the high-energy demands of pregnancy, mandatory maternal physiological changes are required to provide a suitable and continuous metabolic supply from the mother to the foetus [1]. Some metabolic adaptations include the switch from accumulating lipids [2–4] to lipolysis [5] accompanied by the development of insulin resistance [6] through gestation (Fig. 1). In addition to metabolic changes, immunological changes are required to prevent maternal rejection of the foetus, which expresses paternal antigens and is referred to as a semi-allograft [7]. It is now well recognised that the immunoinflammatory processes initiated in response to the blastocyst, embryo then foetus are essential to every stage of pregnancy ensuring successful implantation, gestation, and finally parturition [8–13]. Cellular metabolism has emerged as a key determinant of immune cell function; the pivotal contribution of this is recognised through the term immunometabolism and the associated burgeoning literature [14–17]. Immunometabolic adaptations are well recognised to alter cytokine production, phagocytosis, proliferation, reactive oxygen species (ROS) production, cell death and other effector function of all immune cell types, and there are plenty of reviews that explore this generally [18, 19] or in disease specific settings especially cancer and cardiovascular disease [20, 21].

Exquisite temporal and spatial control of immunoinflammatory and metabolic pathways ensure a successful pregnancy that delivers a healthy baby at full term. Disrupted immunoinflammatory and metabolic homeostasis consequently underpin a host of adverse obstetric outcomes from miscarriage [22] to preeclampsia [23–25], gestational diabetes [26–28], and preterm birth [11]. Understanding this interplay in adverse and healthy pregnancy serves two key purposes. With regards to adverse pregnancy, elucidating underpinning mechanisms will clearly have positive impacts on prognosis, diagnosis, intervention, and treatment for better pregnancy outcomes. In parallel, illuminating the normal immunometabolic adaptations that occur in healthy pregnancy will increase our knowledge of the breadth of immune plasticity possible in humans and enable us to harness this therapeutically for multiple diseases, including those outside of the setting of pregnancy. For example, clinical improvements in rheumatoid arthritis and multiple sclerosis are common in pregnant women, especially in the third trimester [29–32], so understanding why this occurs could reveal shared mechanistic processes in these diseases as well as targets for disease improvement in all people with these autoimmune diseases.

Abnormal and/or excessive fat accumulation with systemic low-grade inflammation to create a health risk characterises obesity [33]. A body mass index (BMI = weight (kg)/height (m)^2^) above 30.0 is classed as obese in accordance with World Health Organisation (WHO) criteria, with those with a BMI ≥ 40.0 classed as morbidly obese. Obesity is a global epidemic, with prevalence rates increasing worldwide in both developing and developed countries. In England 2019, 29% of adults and 18% of children were classed as obese [34]. Obesity leads to serious comorbidities such as type 2 diabetes mellitus (T2DM), reproductive dysfunction, and cardiovascular disease. In England 2019, 10,660 and 711,000 hospital admissions were directly caused by obesity or where obesity was a factor, respectively [34]. The majority of these hospital admissions were female (74% and 66%, respectively) [34]. In women of reproductive age the prevalence of obesity is between 20% and 28% [34]. Obesity is considered a chronic inflammatory disorder with reciprocal effects on adipose tissue (AT) and immune cells that extend beyond AT into the peripheral circulation and other tissues such as the liver [35]. The interplay between immune and metabolic pathways regulates inflammatory cascades typically initiated by pathogen- and danger-associated molecular patterns (PAMPs and DAMPs) and obesity is now well recognised to perturb a diverse range of inflammatory cascades through effects on these pathways. This is best evidenced by infection complications and increased risk of vaccine failure [36, 37]. Examples include a higher risk for viral and bacterial infections along with secondary infections such as sepsis (women only) and community-acquired pneumonia [38, 39]. Immunometabolic derangement of key immune cells such as AT macrophages is well described is animal models of obesity and our understanding of similar processes in humans is growing [40]. Given the apparent importance of immunometabolism in immune plasticity in pregnancy, the epidemic of obesity recognised for the bi-directional interaction between the immune system and metabolism [40] poses a particular risk to the maternal-foetal dyad. Here we will review the metabolic changes that occur in pregnancy, explore how they link to immune function at this time and consider the effects of obesity on these. Our focus is inflammation and the contribution of monocytes and macrophages given that obesity is a low-grade chronic systemic inflammatory and balancing pro- and anti-inflammatory processes is critical to pregnancy success.

## Pregnancy and immune plasticity

The maternal immune system undergoes dynamic adaptation to pregnancy. This is now well recognised to occur at the materno-foetal interface which is composed of foetal-derived trophoblast cells of the placenta and chorionic membrane juxtaposed to the maternal decidual cell populations in intimate contact with the placenta proper and the foetal-placental membranes but also maternal blood-borne cells within the intervillous space of the placenta as it develops. Systemic changes in maternal immune function also occur with extracellular vesicles of various size from the placenta present in the maternal circulation offering a further site of materno-foetal interaction [41]. Together, this ensures immunoregulatory processes that prevent rejection of the foetus yet maintain protection against pathogens, with the underpinning immune plasticity described as an immune clock [42]. Additional to changes in immune cell subsets both within the uterus and systemically that are extensively reviewed elsewhere [7, 43], both pro- and anti-inflammatory pregnancy stage-specific changes occur as a healthy pregnancy progresses [44]. Contrary to past perceived detrimental effects of these responses, provoking inflammatory cascades within the uterus and systemically facilitates pregnancy. At the maternal-foetal interface, pro-inflammatory M1 macrophages are dominant in the first stage of pregnancy supporting implantation and contributing to the high levels of tumour necrosis factor-alpha (TNFα), interleukin (IL)-6, and IL-1β observed [8, 9]. With the emergence of the developing placenta, an anti-inflammatory M2 milieu becomes dominant supporting trophoblast invasion and vascular remodelling, and includes secretion of mediators such as IL-10 and TGFβ [10]. A recent study has further highlighted the critical homeostatic regulatory role of macrophages in a successful pregnancy, with murine models exhibiting insufficient maternal CD11b+ myeloid cells resulting in neonatal death and preterm birth [45]. The expansion of paternal antigen-specific Treg cells and a switch to a Th2 profile with the secretion of IL-4 and IL-10 assists in the prevention of allograft rejection [46, 47]. During the last two trimesters of pregnancy, maternal systemic immunity favours an anti-inflammatory innate phenotype [48]. A number of studies demonstrate that plasma levels of many pro-inflammatory cytokines (e.g. IL-18, TNFα, CCL2, CXCL10, IL-6) are decreased in pregnancy, whereas mediators with immunomodulatory and anti-inflammatory properties (e.g. IL-1 receptor agonist (RA), soluble (s) TNF-receptor II, and sTNF-RII) are elevated [48, 49]. Consequently, perturbed levels of pro-inflammatory cytokines are associated with adverse obstetric outcomes. For example, systemic elevation of IL-6 is associated with preeclampsia [24]. The induction of labour is then associated with a pro-inflammatory state, with pro-inflammatory macrophages infiltrating the decidua to promote uterine contractions [11]. Prior to this, in preparation for delivery, elevated levels of predominantly pro-inflammatory cytokines are observed (e.g. IL-1β, IL-1α, IL-6, and IL-8) [12, 13].

As key contributors to inflammatory processes in both pregnancy and obesity, monocytes and macrophages will be the focus of our considerations hereon. Monocytes are key innate effector cells and precursors of macrophages so have a central role in inflammation. They can be characterised as classical CD14^++^CD16^−^ monocytes that account typically for 90–95% of total monocytes, intermediate CD14^++^CD16^+^ monocytes that account for < 1%, and non-classical CD14^+^CD16^++^ that account for 5–10%. While classical monocytes are more prevalent, non-classical monocytes are more pro-inflammatory [50] and are elevated in inflammatory states such as Gram-negative sepsis and rheumatoid arthritis [51–53] and are increased with pregnancy [54]. Monocytes are also typically more activated during pregnancy when compared with non-pregnant women evidence by increased expression of the activation markers CD14, CD64, and CD11b [55, 56]. Monocytes in pregnancy also produce more oxygen-free radicals [55], with elevated oxidative burst [56], but their ability to produce cytokines in comparison to those from non-pregnant women seems to depend on the cell stimulation used [57–61]. Monocytes in pregnancy also appear primed to express IL-12, a type 1 cytokine [60], further implicating monocytes as being in a state of heightened activation in pregnancy.

Despite advances in our understanding of macrophage heterogeneity, they are typically classified into two subsets: M1, typically IFNγ or lipopolysaccharide (LPS) conditioned, that are classically activated and pro-inflammatory; and M2, typically IL-4 conditioned, that are alternatively activated and anti-inflammatory. Macrophages are critical to the development of the placenta and we have recently reviewed their plasticity in reproduction [62]. They are abundant in the decidua where they contribute to the local tissue microenvironment and in the cycling endometrium in not pregnant women where, due to their fluctuating numbers during the menstrual cycle, they are thought to be regulated by hormonal signalling [63]. Decidual macrophages, which are maternal in origin, have a vital role in implantation producing factors to promote angiogenesis and tissue remodelling and the clearing of apoptotic cells to prevent release of pro-inflammatory products in preparation for trophoblast invasion [64, 65]. The balance of decidual M1/M2 macrophages is critical to successful pregnancy with imbalances linked to complications such as preeclampsia and miscarriage [22, 23, 25, 62], with M1-like polarisation linked to spontaneous preterm labour [66]. Decidual M1 macrophages are associated with the onset of labour as during the third trimester they infiltrate the decidua which can occur prematurely in preterm delivery [11]. Studies have shown, however, that mapping the conventional M1/M2 paradigm to decidual macrophages is challenging, due to their divergent phenotype in the first and second trimester [10, 67]. It is the accumulation of the M1 subset of macrophages in AT which is associated with obesity and confers local inflammation and insulin resistance [68]. AT macrophages are activated via TLR4 by LPS which is elevated in obesity, potentially due to alterations in the gut microbiota, dubbed metabolic endotoxemia [69]. These activated macrophages recruit monocytes and other leukocytes into the AT via expression of chemokine receptors such as CCR2 and CCR5, as well as secretion of chemokines and cytokines. Due to the difficulty in reconciling macrophage phenotypes in humans in vivo, future studies should consider unsupervised or high-dimensional approaches such as flow or mass cytometry or single-cell genomics. Advances in multiplex immunohistochemistry and digital spatial profiling that can be conducted on tissue sections likely to revolutionise our understanding of human reproduction biology.

## Pregnancy and metabolism

Pregnancy has been described as a diabetogenic state due to increased maternal glucose production, glucose intolerance, and insulin resistance [70]. This is to ensure a continuous supply of glucose to the foetus for growth and development. To fuel her own energy demands over the term of her pregnancy, the mother transitions from an anabolic state in which she stores lipids, to a catabolic state of breaking them down via lipolysis [71]. This is evidenced by a decrease in maternal adipose tissue deposits and an increase in levels of postprandial-free fatty acids (FFAs) in late pregnancy [72]. To meet these energy demands, appetite and therefore food intake is increased, and activity is reduced. Over the course of pregnancy, approximately 30 000 kcal of energy reserves are established, ~3.5 kg of fat is accumulated and the mother, foetus, and placenta synthesise 900 g of new protein. The net energy cost of reproduction has been estimated to be between 75 000 and 85 000 kcal [73] which is equivalent roughly to running 850 miles. During early pregnancy, glycogen and protein synthesis in muscle is increased, whereas in the liver glycogenolysis is increased and glycolysis is decreased. Insulin levels are increased at the start of pregnancy but insulin resistance starts as early as 12–14 weeks of gestation [2]. During the third trimester, there is an increase in maternal ketone production, intestinal dietary fat absorption is increased, and, in the liver, gluconeogenesis is decreased [74]. Maternal protein storage is also increased during the first half of pregnancy, with these utilised more economically than in the non-pregnant setting in the second half of pregnancy [75, 76].

In the first two trimesters, heightened lipogenesis allows for the accumulation of maternal adipose tissue deposits which are catabolised in the last trimester [4]. The synthesis of triglycerides increases by 40% by 18 weeks of gestation, and by 250% by term [4, 77]. High-density lipoprotein (HDL), very-low density lipoprotein (VLDL), and low-density lipoprotein (LDL) are part of a family termed lipoproteins which are a combination of various types of fats and proteins. They are necessary for the transport of cholesterol and triglycerides through the blood. In the first 24 weeks of gestation, HDL levels increase progressively before decreasing until 32 weeks where they plateau [4]. LDL levels undergo a small decrease during the initial stages of pregnancy before rising steadily [4]. During the second and third trimester, VLDL levels increase threefold. These net increases of lipoprotein levels are due to cholesterol levels being 50% higher in late pregnancy in comparison to pre-pregnancy [4, 77].

Metabolic processes during pregnancy are influenced by hormones such as oestrogen, human placental lactogen, progesterone, and potentially the satiety hormone leptin. These hormones can alter the action of insulin and the utilisation of glucose, leading to the observed diabetic state which pregnancy has been likened to. This can also induce changes in the metabolism of proteins and lipids, as well as increase amino acid and glucose availability for the foetus, whilst meeting the maternal needs by providing FFAs as an alternative energy substrate to maintain homeostasis. The importance of metabolic adaptation in pregnancy is evidenced by the ability to use measures of circulating metabolites to predict gestational age and time to labour onset [78].

### Leptin

Leptin signalling plays an important role during pregnancy and obesity independently and with known effects on leukocyte function needs to be considered as a mediator of any interaction between obesity and pregnancy. Leptin is increased with both obesity and pregnancy, but in pregnancies with obesity maternal leptin is actually decreased in comparison to lean pregnant women [79]. Leptin classically controls food intake by interacting with leptin receptor (OB-R; CD295) in the brain [80] and leptin resistance leads to over-indulgence and further fat storage [81]. Leptin is also responsive to other hormones, such as insulin and cortisol which induce upregulation [82] while catecholamines downregulate leptin [83]. Leptin expression is regulated by a multitude of other factors such as TNFα which increases leptin secretion [84] and by glucose and fatty acids [85]. Leukocytes also express OB-R so leptin can have effects on both innate and adaptive immune features. It can activate innate immune cells (e.g. monocytes, NK cells, neutrophils), improve their survival by downregulating apoptosis and upregulating cell proliferation and chemotaxis [86], and enhance their functions such as cytotoxicity (NK cells), phagocytosis (macrophages) and cytokine release [87]. Leptin also favours pro-inflammatory CD16^+^ non-classical monocytes [88] and induces the proliferation of most adaptive immune cell types, except Tregs, and improves their responsiveness to chemotactic signals, while promoting a switch towards pro-inflammatory Th1 [87]. Overall, leptin promotes a pro-inflammatory phenotype.

Leptin is essential for some reproductive functions, with leptin injections improving the likelihood of conception in infertile mice [89] and leptin initiating the onset of puberty in human females [90]. In normal pregnancy, elevated levels of leptin occur due to not only AT accumulation but also placental production [91]. Leptin has been described as having various gestational regulatory roles including placental angiogenesis, nutrient transport, and immunomodulation [92]. Central leptin resistance develops as a normal part of pregnancy during the second trimester [93] with maternal obesity linked to the development of placental leptin resistance [94]. Foetal growth is supported through central leptin resistance mechanisms in healthy women, but placental leptin resistance in women with obesity adversely affects feto-placental growth and development [95]. It also seems that central leptin resistance mechanisms might differ in healthy versus pregnant women with obesity and this has been reviewed elsewhere [95]. Very little is known about the immunomodulatory effects of leptin in pregnancies with maternal obesity, although it has been suggested that pre-gravid high circulating leptin levels in women with obesity prime the placental inflammation observed in these pregnancies [96].

## Pregnancy and immunometabolism

**Figure 2:**
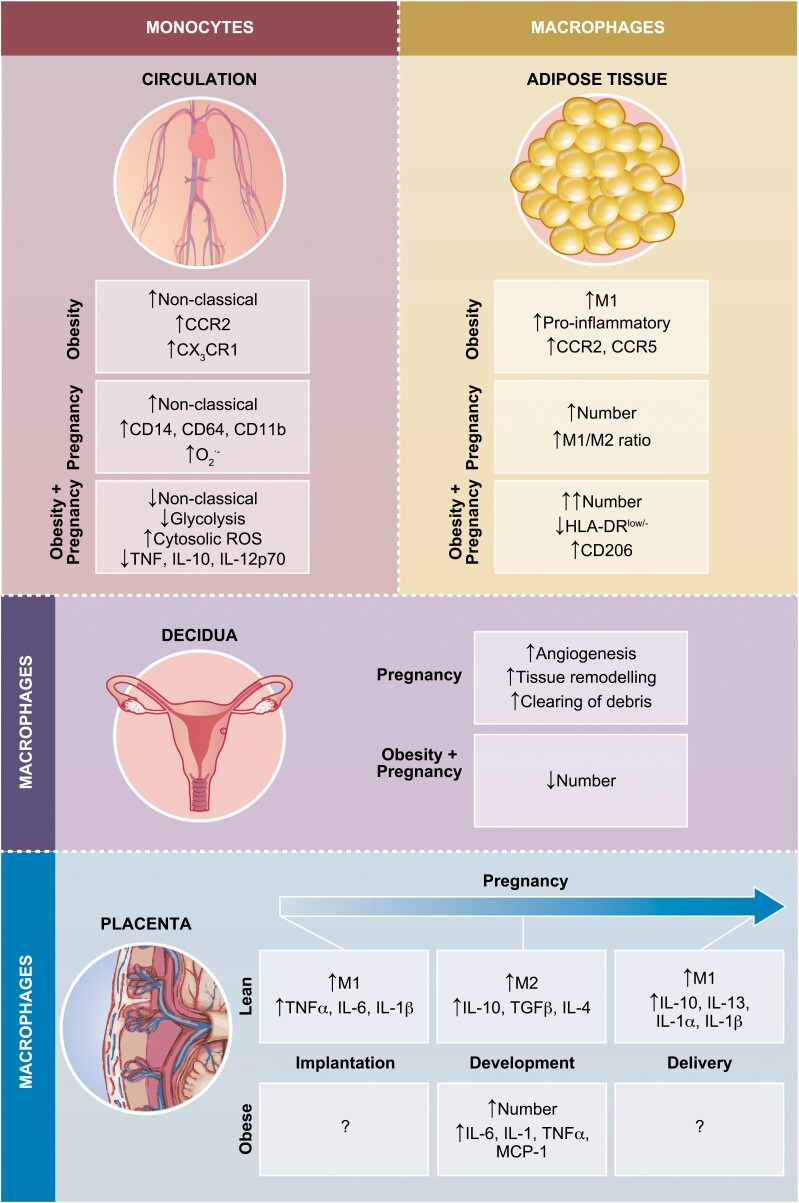
A summary of the effects obesity has on monocytes in the blood and macrophages in adipose tissue and in the placenta. Monocytes: in comparison to lean, obesity is associated with an increase in non-classical (CD14^dim^CD16^+^) monocytes [212], which are thought to be more pro-inflammatory, as well as an increase in the expression of CCR2 and CX_3_CR1 [50, 51]. Pregnancy is also accompanied by an increase in non-classical monocytes. Monocytes in pregnancy also have increased expression of CD14, CD11b and CD64, as well as increased superoxide production [53]. Adipose tissue: obesity is associated with an increase in M1 macrophages, inducing a more pro-inflammatory environment [213]. These macrophages also have elevated expression of chemokine receptors CCR2 and CCR5. During a healthy pregnancy, the number of total macrophages increases in adipose tissue and there is an increase in the M1/M2 ratio. The effect obesity has on pregnancy shows that the number of macrophages is further exacerbated, and also increases the induction of apoptosis. Surface markers on these macrophage changes: lower HLA-DR^low/−^ and elevated CD206 [22]. Decidua: during a healthy gestation, decidual macrophages promote angiogenesis, tissue remodelling and the clearance of apoptotic cells [68, 214]. Very little is currently understood about decidual macrophages in an obese environment, but their overall numbers are reduced [23]. Placenta: throughout a healthy pregnancy placental macrophage undergo many changes. At implantation, M1 macrophages are dominant and contribute to the high levels of TNFα, IL-6 and IL-1β observed [215, 216]. As pregnancy develops, a switch occurs so that M2 are the dominant macrophage phenotype, along with the increased production of IL-10, TGF-β, and IL-4 [217]. At delivery, the macrophages switch back to an M1 phenotype which coincides with elevated production of IL-10, IL-13, IL-1α, and IL-1β [8–10]. Little is known about the effect obesity has on macrophages in the placenta but it has been shown that there are more placental macrophages in obesity, and an increase in pro-inflammatory cytokines IL-6, IL-1, TNFα, and MCP-1 [96].

Immunometabolism is a burgeoning field with the main tenet being that the metabolism of immune cells impacts function (Fig. 2) [14] and we have summarised elsewhere how changes in metabolism with pregnancy might impact T-cell function [97]. Upon activation, many leukocytes appear to prefer glycolysis over oxidative phosphorylation: while less adenosine triphosphate (ATP) is produced per molecule of glucose, glycolysis provides more rapid ATP production along with key biosynthetic intermediates, supporting a rapid effector response [14]. This is evidenced by the elevated appetite for glucose of activated macrophages and CD4+ T cells [15, 16] and the inhibition of macrophage activation in vitro and suppression of inflammation that occurs in vivo after inhibition of glycolysis [98, 99]. Glycolysis is initiated more rapidly via upregulation of enzymes involved in the pathway, whilst increasing oxidative phosphorylation (OXPHOS) is a much slower process requiring mitochondrial biogenesis. Glycolysis at higher rates also enables production of biosynthetic intermediates to support cell growth and proliferation. However, other ATP and metabolite generating pathways do have a role in immune function. For example, the differentiation of macrophages and the production and maintenance of memory CD8+ T cells require fatty acid oxidation (FAO) [14].

Our focus in this review is monocytes and macrophages so immunometabolism of these will be a critical determinant of their function in both pregnancy and obesity. Monocyte metabolism has been described mostly in tandem with macrophages which has been well reviewed elsewhere [100, 101]. From the little information about monocytes that we have already the key message seems to be that the metabolic programmes which govern their function are unique to individual stimuli and there is not a “one size fits all” switch to glycolysis [102]. There is a rapid increase in glycolysis in response to the TLR4 ligand LPS but this is not seen with the TLR2 ligand P3C (Pam_3_CysSK_4_) or alternative bacterial lysates [102]. To retain their phagocytic and cytokine activity in response to P3C, increased OXPHOS by monocytes is necessary [102]. Mitochondrial morphology might contribute to these differences, as larger mitochondria were observed in the monocytes stimulated with LPS in comparison with P3C after 24-hr stimulation [102].

Early evidence of immunometabolic changes with pregnancy is emerging and includes observations such as reduced pyruvate kinase expression in neutrophils and lymphocytes in pregnancy [103], and reduced glycolytic capacity and basal glycolysis but elevated bioenergetics health index in peripheral blood mononuclear cells of pregnant women [104]. Cyclic ADP ribose hydrolase (CD38), a glycoprotein found on the surface of many leukocytes, provides the best example so far of the potentially diverse effects of immunometabolism on pregnancy. CD38 is a multi-functional ectoenzyme which catalyses reactions that produce cyclic ADP-ribose and nicotinic acid adenine dinucleotide phosphate (NAADP) from NAD+ and NADP+, respectively, and has been postulated to have critical roles in pregnancy. Soluble CD38 (sCD38) from seminal fluid has a vital role in the establishment of maternal immune tolerance in mice [105]. sCD38 promotes the development of forkhead box P3^+^ (Foxp3^+^) Treg cells and uterine tolerogenic DCs to prevent rejection of the allogeneic foetus, with knockout models showing that pregnancy loss occurs [105]. CD38 is also more highly expressed on NK cells during pregnancy where it is postulated to have role in enhancing their ability to combat-infected cells [106]. This clearly needs elaborating given the contradictory effects on mitochondrial function attributed to CD38 outside of pregnancy. For example, in malignant myeloma cells, CD38 is required for oxidative phosphorylation and intercellular mitochondrial transfer [107], yet studies in aging have found that CD38 levels are inversely proportional to mitochondrial function [108]. Elevated expression of CD38 on NK cells could suggest reduced reliance on mitochondrial respiration, and therefore further investigation into the link between cellular metabolism and function is warranted. The normal immunological adaptations that accompany pregnancy are vital to ensuring pregnancy success so compromise of these could underpin adverse pregnancy outcomes associated with maternal obesity. CD38 expression on monocytes and macrophages is induced in inflammatory conditions [109] and a decrease in the expression of CD38 correlates with suppression of adipogenesis and lipogenesis in adipose tissue in mouse models [110]. Inhibitors of CD38, such as the flavonoid apigenin from foods such as parsley, have shown beneficial effects in tackling obesity in animal models [111]. In these models, elevated cellular levels of NAD^+^ are beneficial, and CD38 knockout increases the NAD^+^ levels and protects against obesity [111].

## Pregnancy and obesity

Obesity has been implicated in impaired reproductive function and, in pregnancy, is associated with increased risk of miscarriage and other adverse outcomes such as gestational diabetes mellitus (GDM) and preeclampsia [112–114]. Maternal obesity also increases the risk of foetal mortality, childhood obesity stemming from macrosomia, and metabolic syndromes for both the mother and the foetus [115–117]. Adverse perinatal outcomes linked to obesity have been associated with increased placental and systemic inflammation, oxidative stress, pre-pregnancy maternal insulin levels and hyperinsulinemia [96, 118–120]. Much of the current research on obesity and pregnancy focuses primarily on the effects on the offspring rather than on the pregnant woman herself beyond the immediate obstetric challenges. Studies of the maternal contribution to the obesity-related milieu in pregnancy also tend to focus on the placenta and to a lesser extent AT, and seldom consider the role of or effect on the peripheral, circulating immune system herein represented by monocytes (see Fig. 3 for summary of all three). This is important because obesity has serious effects on the immune system outside of pregnancy with down-regulated immune responsiveness best evidenced by infection complications and increased risk of vaccine failure [36, 37]. Specific examples include a higher risk for viral and bacterial infections along with secondary infections such as sepsis (women only) and community-acquired pneumonia [38, 39]. Obesity is also an indicator for severity of COVID-19 symptoms, with high levels of C reactive protein (CRP) indicating strong critical illness risk [121].

Obesity commonly induces insulin resistance by chronic activation of inflammatory pathways and heightened infiltration of immune cells into metabolic tissue. This leads to impairment of glucose and lipid homeostasis altering nutrient availability and cellular metabolism. TLR signalling has a role in controlling immunometabolism with saturated and polyunsaturated fatty acids acting via TLR4 to induce immunomodulatory effects. In high saturated fat fed mice, TLR4 deficiency protects the mice from obesity [122, 123]. Palmitate, present in high concentrations in the circulation of similarly fed mice, activates the inflammasome and induces macrophage secretion of IL-1β and IL-18 [124]. Palmitate levels are elevated in obese compared with lean pregnant women [125] and palmitate activates the nucleotide-binding oligomerisation domain-like receptor family pyrin domain containing 3 inflammasome in the placenta for elevated IL-1β secretion [126]. Increased foetal growth is often correlated with maternal obesity; rat models have shown that placental insulin, IGF-1, mammalian target of rapamycin (mTOR) and leptin signalling pathways are more highly activated in maternal obesity, stimulating increased amino acid transport across the placenta and contributing to elevated foetal growth [127]. AMPK and mTORC1 are nutrient sensing pathways which highly influence macrophage polarisation, suggesting that obesity-associated alterations in metabolite signals induce the accompanying altered immune function described but this has not been studied in pregnancy. Mitochondrial dysfunction and deficiencies have been linked with obesity [128]. Adipocytes in obese AT have reduced mitochondrial oxidative capabilities, lowered mitochondrial biogenesis, and downregulated OXPHOS proteins [129–131].

Insight into the possible effect of obesity on immunoinflammatory processes in pregnant women can be provided by the many studies of the general adult population. Excessive leukocyte infiltration in adipose tissue occurs with obesity and contributes to the superfluous production of cytokines, adipokines, and other mediators that drive the characteristic low-grade systemic inflammation [132–134]. Macrophages are postulated to be the primary initiator of this, in turn recruiting other cell types such as monocytes, T cells and B cells, leading to further inflammation. Eosinophils in particular have garnered attention in recent times for their homeostatic roles in AT [135] but consideration of these is beyond the scope of this review. The products of this inflammatory environment are measurable in the circulation such as increased levels of IL-6 and leptin [136, 137]. Obesity has been linked repeatedly to altered peripheral blood leukocyte function at least in the not pregnant population with PBMCs from obese individuals contributing to the systemic chronic low-grade inflammation with their heightened pro-inflammatory phenotype [138]. Inflammation-related adipokines such as osteopontin, lipocalin-2, chitinase-3-like protein 1, and chemerin gene expression are also all upregulated in PBMCs from obese individuals [139].

While the focus of this review is the effects of maternal obesity on monocytes and macrophages obesity in the general population is also recognised to negatively affect the function of multiple lymphocyte populations. This ranges from suppression of T- and NK-cell function – including reductions in cytotoxicity, IFNγ production, and expression of perforin and granzymes [140] – and altered B-cell activity that manifests as reduced class-switching and immunoglobulin activity [141]. Maternal obesity diminishes the numbers of uterine resident NK cells and alters their contribution to extracellular matrix remodelling and growth factor signalling to compromise trophoblast survival and spiral artery remodelling [142]. A reduced CD8+ T-cell count in peripheral blood with obesity in both the general population [143] and pregnant women [144] has been described and possibly links to their accumulation in adipose tissue which, from mouse models, precedes that of macrophages [143]. The cytokine producing capacity of T cells also changes with obesity in the general population and obesity-associated inflammation is in part driven by a shift to Th1 and Th17 which is thought to be mediated by leptin [145]. Th1 and Th17 cytokines such as TNF and IFNγ are detrimental to pregnancy [146]. Conversely, a Th2 and regulatory T-cell (Treg) dominated environment is considered essential to pregnancy success [146]. We do not yet know how maternal obesity impacts this critical cytokine balance but maladaptation of adaptive immune processes could very much underpin obesity-associated adverse obstetric outcomes with upregulation of Th1 described in GDM [147]_._

The non-classical subpopulation of monocytes is increased with obesity [148] and this is accompanied by functional differences in monocytes. As for heterogenous PBMCs, the response by monocytes also favours a pro-inflammatory phenotype through heightened provision of cytokines such as IL-1β and RANTES upon LPS stimulation [149]. Non-classical and classical monocytes can also be identified by chemokine receptor expression as being CX_3_CR1^high^/CCR2^low^ or CX_3_CR1^low^/CCR2^high^, respectively. CCR2 is elevated on total monocytes in obese individuals, and more specifically on classical and intermediate monocytes [149, 150], along with increased intrinsic migratory capacity, potentially due to this elevated CCR2 expression [150]. Higher expression of CX_3_CR1 by all three subsets suggests an inclination to recruitment by CX_3_CL1-secreting AT [149]. If these chemokines receptors are found to be exacerbated further during pregnancy with maternal obesity, this might suggest that CCR2-mediated recruitment of maternal monocytes is responsible for the accumulation of pro-inflammatory placental macrophages.

Like pregnancy, there appears to be very little research surrounding specific immune cell bioenergetics in obesity although the spare respiratory capacity of monocytes has been negatively correlated with percentage body fat [151]. A study investigating the effect of the bioenergetic function of peripheral monocytes in obese and lean women with HIV illustrated that the monocytes in obese infected women had impaired bioenergetic health (reduced basal and maximal oxygen consumption rate as well as decreased bioenergetic health index) in comparison to lean infected women [152].

## Pregnancy, immunometabolism, and obesity

Inflammatory alterations which occur normally in a healthy pregnancy may be exacerbated with maternal obesity. Further elevated circulating maternal IL-6 and CRP levels have been reported in pregnant women with obesity [96]_._ This has consequences for the foetus with increased leptin and IL-6 in umbilical cord plasma and acquisition of insulin resistance in utero of new borns of obese mothers [153]. FFA and AT deposits are in excess with maternal obesity and elevated foetal exposure to FFA correlates with neonatal adiposity and potential future obesity of the child [154]. However, the impact of obesity on maternal immunometabolism is largely unknown with also little known about the effects of maternal obesity on monocytes and macrophages. Given the dramatic effects on monocytes of either obesity or pregnancy alone further consideration of the combined effect is certainly warranted. A recent study has shown that at 37 weeks of pregnancy, monocytes from pregnant women with obesity have reduced extracellular acidification rate (glycolysis indicator) at baseline and following LPS and glucose injections [155]. This supports maladapted immunometabolism with maternal obesity and is accompanied by fewer monocytes performing phagocytosis and reduced cytokine response to LPS. The consequences of this effect on monocytes with maternal obesity, however, cannot be concluded without data on how the monocytes adapt metabolically in a healthy pregnancy, and the effect on the monocytes at different trimesters.

Maternal obesity has been associated with changes to macrophages in the placenta and decidua and these have been reviewed elsewhere [156]. Briefly, increased inflammation including heightened gene expression of IL-6, IL-1, TNFα, and MCP-1 linked to increased number of macrophages have been described to occur in the placentas of obese versus lean pregnant women [96]although there are some conflicting findings regarding changes to the number of placental macrophages with maternal obesity [157]. In contrast, decidual macrophage levels are reduced in obese compared with lean women [158]. These changes have been postulated to be linked to placental dysfunction and foetal programming of later child health [96]. AT macrophages in pregnant women are not well studied due to the difficulty obtaining AT from pregnant women. One study using visceral adipose tissue collected from the omentum during caesarean section found that pregnant women who were overweight or obese had adipocyte hypertrophy and changes to specific populations of macrophages in AT (e.g. lower number of resident HLA^-^DR^low/−^ macrophages, higher expression of CD206 on resident and recruited macrophages, and elevated CD11c expression on resident HLA-DR^+^ macrophages) [159].

The fatty acid translocase (FAT) CD36 has been implicated in various obesity-related complications. For example, CD36 is normally highly expressed in AT and is up-regulated further in obesity and T2DM [160]. Animal studies support a role for CD36 in impaired glucose tolerance and insulin signalling in obesity as well as macrophage infiltration into AT and overproduction of inflammatory cytokines and chemokines [161]. While much less studied in humans, monocytes of obese non-pregnant women aged 25–45 years showed significantly decreased expression of CD36 compared with lean individuals [162] but such studies have not been extended to pregnant women. If this phenotype does extend to monocytes from pregnant women, it suggests that the monocytes would be less capable of combating PAMPs or DAMPs, with decreased ability to take up fatty acids, and reduced capability to partake in non-opsonic phagocytosis (which is another role of the scavenger receptor [163, 164]). While little is known about changes in expression of nutrient transporters systemically in pregnant women either with or without obesity the contribution to placental function in health and disease is recognised increasingly and there are also some studies in AT of pregnant women [165]. CD36 is expressed by the placenta supporting responsiveness to lipoproteins in maternal plasma [166]. Upregulation of other transporters in the placenta such as long chain neutral amino acid transporter (LAT1; CD98) and sodium-coupled neutral amino acid transporter (SNAT) 2 has been linked with foetal macrosomia secondary to maternal obesity in murine models [127]. Glucose is transported across the cell plasma membrane via glucose transporters (GLUT). Different GLUTs have different distributions and functions. GLUT1 is expressed on all cells but is expressed most highly in erythrocytes [167] and barrier tissue endothelial cells [168]; GLUT3 is expressed predominantly in neurons [169] and the placenta [170] and GLUT4 in AT [171] and striated muscle [172]. Glucose intolerance is observed commonly in both pregnancy and obesity independently of each other, and in rat models impaired glucose tolerance is further exacerbated in obese pregnancy [173]. GLUT1 is naturally overexpressed in macrophages in obesity, and this induces a pro-inflammatory response dependent on glycolysis and ROS [174]. How these pathways support the immune functions of placental macrophages and trophoblast cells remains to be determined although metabolic disarray of trophoblast cells linked to GDM is a common finding [175, 176].

## Obesity is a risk factor for adverse pregnancy outcomes

### Gestational diabetes mellitus

Indeed, perhaps the greatest evidence of a link between metabolism and immunoinflammation in pregnancy and a bi-directional interaction with obesity is provided by adverse pregnancy outcomes such as GDM. GDM is characterised by varying severity of glucose intolerance, with onset or recognition during pregnancy and while it usually resolves postpartum [177]it is a risk factor for later development of T2DM [178]. In the not pregnant setting, obesity induces insulin resistance and T2DM through the release of cytokines such as TNFα and IL-6 [179, 180]. GDM can lead to adverse obstetric outcomes including preeclampsia, birth trauma, and neonatal hypoglycaemia; the offspring have increased risk of developing metabolic and cardiovascular disorders [181, 182]. Obesity, ethnicity, age, and family history of diabetes are strong risk factors for GDM. Acceleration of the insulin resistance that emerges naturally as normal pregnancy progresses (Fig. 1), driven by lifestyle and genetic factors, is potentially what happens in GDM [183, 184]. This could have profound effects on immunoinflammatory phenotypes and thereby disease development and progression if immunometabolism is indeed a key driver of changing immune-cell function in pregnancy. While not extensively studied there are changes in maternal systemic immunity with GDM including upregulation of the Th1 phenotype which alters the Th1/Th2 ratio in comparison to a normal pregnancy [147]. A recent review [185] has described the current understanding of the effect of GDM on maternal peripheral leukocytes highlighting pro-inflammatory effects mostly evidenced by increases in NK cells [186] and activated T cells [187]. However, few studies investigate changes in immune function prior to or in the early stages of GDM and there is a real shortcoming of functional studies assessing the effects of GDM on maternal immunometabolism and the immune response. The placenta has tended to be the focus of GDM investigations and placental changes with GDM include elevated oxidative stress and foetal thrombosis [188, 189]; a recent review aptly describes the pathophysiology of GDM [190]. With regards to immune-cell populations, an increase in NK cells in the placenta of GDM mothers has been reported [186]. Reports of effects of GDM on placental macrophage numbers and function are conflicting. Several studies have reported a more pro-inflammatory phenotype [27, 28, 191], whilst others show that the M2 phenotype is maintained [192] but the contribution of alterations to immunometabolic process remains unknown.

### Preeclampsia

Preeclampsia is a hypertensive disorder of pregnancy accompanied by proteinuria which can cause severe effects for mother and foetus. In developed countries, 16% of maternal deaths are caused by hypertensive disorders [193]. The overall risk of preeclampsia is increased by obesity, with the risk increasing as BMI increases and weight loss reducing this risk [194, 195]. It has been hypothesised that obesity is a contributing factor to hypertension via a myriad of mechanisms including: oxidative stress caused by reducing nitric oxide, increased release of free fatty acids from adipocytes, and elevated angiotensinogen [196]. CRP, IL-6, TNFα, and leptin, as discussed above, are found to be elevated in individuals with obesity and are also strongly associated with the development of preeclampsia [197–199]. Abnormalities in placental growth and function drive the pathophysiology of preeclampsia, with changes such as reduced perfusion and oxidative stress inducing a hypertensive environment [200]. As in obesity, both circulating and decidual neutrophils [201, 202] and monocytes [203, 204] have been described to be excessively activated in preeclampsia, with the more pro-inflammatory monocyte subsets (non-classical and intermediate) being elevated even further than they are in a healthy pregnancy [54]. T cells, NK cells and DCs have also been shown to have an altered response in preeclampsia, with a tendency towards being pro-inflammatory rather than supporting the immune tolerance [205–207].

## Conclusion and future outlooks

Much of the effort to understand the contribution of cellular metabolism to immunoinflammatory phenotype and function in pregnancy lies in recent reviews that have covered topics such as the potential role of lactic acid in the uterine microenvironment especially during early pregnancy [208], the potential wider roles of immunometabolism at the maternal-foetal interface [209, 210], the possible contribution of immunometabolism of macrophage fate and function in the pregnant and not pregnant uterus [62], maternal T-cell plasticity in pregnancy [97] and the related area of neonatal immune function [211]. It seems we are on the cusp of greater than ever before understanding of the mechanisms that drive dynamic immune adaption in pregnancy by considering this from the perspective of immunometabolism especially how this links with pregnancy hormone and foetal antigen-driven reactivity. Deep immune phenotyping, other single-cell analysis strategies and spatial profiling of placenta (and adipose tissue) are well placed to support such an analysis.

## Data Availability

Not applicable.

## Abbreviations

AT: adipose tissue
ATP: adenosine triphosphate
BMI: body mass index
FAO: fatty acid oxidation
FAT: fatty acid translocase
FFA: free fatty acids
GDM: gestational diabetes mellitus
GLUT: glucose transporters
HDL: high-density lipoprotein
LDL: low-density lipoprotein
LPS: lipopolysaccharide
mTOR: mammalian target of rapamycin
OXPHOS: oxidative phosphorylation
P3C: Pam3CysSK_4_
ROS: reactive oxygen species
sCD38: soluble CD38
T2DM: type 2 diabetes mellitus
VLDL: very low density lipoprotein
